# The impact of physiological oxidants on triggering amyloid formation of the cell cycle regulator protein p16^INK4A^

**DOI:** 10.1002/pro.70723

**Published:** 2026-07-15

**Authors:** Briana R. Smith, Nicholas J. Magon, Aakriti Sethi, Shelby G. Gray, Sarah G. Heath, Vanessa K. Morris, Christoph Göbl

**Affiliations:** ^1^ Mātai Hāora – Centre for Redox Biology and Medicine, Department of Pathology and Molecular Medicine University of Otago Christchurch New Zealand; ^2^ School of Biological Sciences University of Canterbury Christchurch New Zealand; ^3^ Biomolecular Interaction Centre University of Canterbury Christchurch New Zealand

**Keywords:** amyloid, cysteine oxidation, hydrogen peroxide, hypothiocyanous acid, p16, peroxymonocarbonate, protein oxidation, redox reaction

## Abstract

The tumor suppressor protein p16^INK4a^ plays a key role in cell cycle control and is frequently inactivated in human cancers. We previously discovered that oxidation of its single cysteine residue induces a structural transition from the functional monomeric form into inactive amyloid fibrils. Here, we investigate whether five physiologically relevant oxidants can trigger the conformational switch of the protein. Using time‐resolved SDS‐PAGE, thioflavin‐T fluorescence assays, mass spectrometry, and electron microscopy, we show that peroxymonocarbonate and hypothiocyanous acid (HOSCN) efficiently induce cysteine‐dependent dimerization with subsequent amyloid formation of recombinant p16. In contrast, hydrogen peroxide, hypochlorous acid, and taurine chloramine are less efficient and predominantly lead to the formation of amorphous aggregates. These findings demonstrate that physiological oxidants differ in their ability to induce amyloid transitions in p16, with potential implications for oxidative stress‐mediated inactivation of this cell‐cycle regulator protein.

## 1 INTRODUCTION

The tumor suppressor protein p16^INK4a^ (p16) plays a crucial role in cell cycle regulation (Liggett & Sidransky, 1998; Serra & Chetty, 2018). The 16‐kDa protein inhibits the cyclin‐dependent kinases 4 and 6, and thereby prevents the phosphorylation of the retinoblastoma (Rb) protein (LaPak & Burd, 2014; Li et al., 2011; Serra & Chetty, 2018; Serrano et al., 1993). Hypophosphorylated Rb tightly binds the E2 promoter binding factor 1 (E2F1), blocking cell cycle progression from G_1_ to S phase, making p16 a key regulator of cell division (Li et al., 2011). p16 is frequently inactivated in a wide range of human cancers (Drexler, 1998; Harms et al., 2016; Jenkins et al., 2011; Nobori et al., 1994; Pinyol et al., 1997; Romagosa et al., 2011). Loss of function of p16 enables precancerous cells to bypass senescence and progress to malignant disease (Harms et al., 2016; LaPak & Burd, 2014; Serra & Chetty, 2018; Witkiewicz et al., 2011). For example, p16 is often inactivated during the progression to invasive disease in pancreatic intraepithelial neoplasia (Hruban et al., 2000). Restoring p16 expression in p16‐deficient solid tumor cell lines reduces cellular proliferation and tumorigenic phenotypes (Gombart et al., 1997), further implicating p16 inactivation as a critical step in malignant transformation.

**FIGURE 1:**
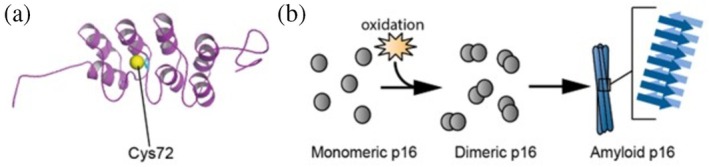
Structure of p16 and scheme of transition into amyloid structures. (a) p16 is a small, α‐helical protein consisting of four ankyrin repeats connected by rigid hairpin loops in a helix‐turn‐helix motif flanked by flexible termini (PDB ID: 2A5E) (Byeon et al., 1998). The critical Cys72 residue is found in the loop connecting the second and third ankyrin repeats; the cysteine backbone is displayed in cyan and the sulfur atom position is displayed as a yellow sphere. (b) Upon oxidation, monomeric p16 forms a short‐lived, homo‐dimeric species that subsequently folds into amyloid (Heath, Gray, et al., 2024; Heath, Naughton, et al., 2024).

The molecular structure of p16 consists of four central α‐helical ankyrin repeats, connected by rigid loop regions, and flanked by highly flexible termini (Byeon et al., 1998; Rocco & Sidransky, 2001; Tang et al., 1999). p16 contains a single cysteine residue (Cys72), the side chain of which is oriented toward the surface (Figure 1a). Cysteine thiols are frequent targets of oxidative modifications, including the formation of disulfide bonds (Winterbourn, 2008). These oxidative modifications can facilitate major structural changes of proteins that impact protein function (van Dam & Dansen, 2020). We have previously shown that oxidation of the single cysteine residue of p16 induces formation of disulfide‐crosslinked homodimers. The homodimeric species subsequently fold into β‐sheet rich aggregates with typical features of amyloid fibril structures (Göbl et al., 2020). Notably, when p16 is in the amyloid state, it is unable to bind to CDK4/6 and inhibit Rb phosphorylation (Heath, Gray, et al., 2024). This suggests that oxidative stress may play a role in p16 inactivation. It is currently unclear which physiological oxidants are able to induce this specific structural change. Several oxidants are present during cellular processes, and they have distinct reactivities and specificities towards different targets (Murphy et al., 2011; Sies et al., 2022; Winterbourn, 2008). For example, peroxymonocarbonate (HCO_4_^−^), an oxidant produced in an equilibrium reaction of hydrogen peroxide with bicarbonate, can be more reactive towards certain proteins than hydrogen peroxide alone (Augusto & Truzzi, 2021; Dagnell et al., 2019; Regino & Richardson, 2007; Richardson et al., 2003; Winterbourn et al., 2023).

Here, we study the oxidation process that facilitates the structural transition from the functional monomeric state of human p16 to the inactive amyloid state by testing five physiological oxidants: hydrogen peroxide, peroxymonocarbonate, hypothiocyanous acid (HOSCN), hypochlorous acid (HOCl), and its amino acid reaction product taurine chloramine (TauCl). We characterized the oxidation and amyloid formation process, including kinetics derived from SDS‐PAGE and Thioflavin‐T (ThT) assays, measurements of oxidation products by mass spectrometry, and amyloid morphology analysis using negative stain electron‐transmission microscopy. Our analysis reveals that the major modification taking place is the oxidation of the cysteine sidechain, with only minor modifications of other amino acids. We find that peroxymonocarbonate and HOSCN are the most efficient oxidants for amyloid conversion, suggesting a physiological role for these oxidants in dramatically modifying the structure and function of p16.

## 2 RESULTS

### 2.1 Characterizing the amyloid formation of p16 by the cysteine‐specific oxidant diamide

p16 is an all‐α helical protein that contains a single cysteine residue (Figure 1a) and forms a transient dimeric species that subsequently aggregates into amyloid structures (Figure 1b). To explore the oxidation‐dependent dimerization of p16 that triggers amyloid formation, we exposed purified p16 to the thiol‐specific model oxidant diamide, which does not lead to any other amino acid modifications (Heath, Naughton, et al., 2024). As a reference experiment, purified protein was treated with a 10‐fold excess (200 μM) of diamide and incubated for multiple time points. The oxidation reaction was then quenched by the addition of *N‐*ethylmaleimide (NEM) and analyzed by SDS‐PAGE (Figure S1a). Upon treatment with diamide, we observed the appearance of a protein band at approximately 30 kDa, corresponding to the expected mass of the p16 homodimer. The intensity of this band increased over time, suggesting a time‐dependent increase in dimer concentration, as reported previously (Heath, Gray, et al., 2024).

After approximately 1 h, the dimer and monomer bands reached equal intensity (hereafter referred to as approximate dimerization half‐time). The p16 dimer band was reduced (R) by the addition of β‐mercaptoethanol (BME) in the SDS‐PAGE sample buffer. To determine reaction kinetics of amyloid fibril formation, we used a Thioflavin T (ThT) fluorescence assay as established previously (Heath, Gray, et al., 2024). The fluorescence signal of the oxidized sample resulted in a typical sigmoidal‐shaped curve reaching a stable plateau and no signal change was observed for the unoxidized control sample (Figure S1b). The amyloid formation half‐time was reached at 103.2 ± 2.6 min after the addition of oxidant, showing that amyloid fibril formation succeeds dimerization of p16, as described previously (Göbl et al., 2020). We further confirmed the amyloid fibril formation of diamide‐treated p16 using negative‐stain transmission electron microscopy, which showed the presence of fibrillar structures when measured 48 h after treatment with diamide (Figure S1c). These experiments serve as a reference for the testing of physiological oxidants.

### 2.2 Homodimerization of p16 is dependent on the type of oxidant

Next, we explored the effect of physiological oxidants on p16, and their ability to induce the formation of the dimeric species. The oxidants used were hydrogen peroxide in the absence (H_2_O_2_) or presence of bicarbonate (H_2_O_2_ + HCO_3_^−^), HOSCN, HOCl, and taurine chloramine. Many of these oxidants are important signaling molecules, for example being produced at sites of inflammation. Hydrogen peroxide is a by‐product of the mitochondrial electron transport chain and can also be produced catalytically by various enzymes (Davies, 2011). It can also react with other molecules to form further reactive species. Recent studies have highlighted the formation of peroxymonocarbonate from H_2_O_2_ and bicarbonate; the latter is ubiquitously produced through metabolic decarboxylation reactions. In some cases, peroxymonocarbonate is a more efficient oxidant than hydrogen peroxide (Regino & Richardson, 2007; Richardson et al., 2000; Richardson et al., 2003; Winterbourn et al., 2023). HOSCN is a thiol‐specific oxidant that is produced by the innate immune system by peroxidase enzymes that catalyze its production from hydrogen peroxide and thiocyanate. It is a major oxidant at inflammatory sites and causes damage to pathogens (Ashby, 2012; Davies, 2021; Skaff et al., 2009) but has also been shown to modify cysteines of proteins and peptides within human cells. HOCl, commonly referred to as chlorine bleach, is a highly reactive oxidant produced during the innate immune response that reacts with almost all biological molecules and proteins (Davies, 2011; Hawkins, 2019; Van et al., 2018). It is produced by peroxidases that oxidize chloride to HOCl using hydrogen peroxide (Kettle et al., 2014). It can be toxic to cells and, through its high reactivity, often reacts with amino acids, such as the highly abundant taurine, to form taurine chloramine (TauCl), attenuating the effect of HOCl toxicity (Sun Jang et al., 2009; Thomas et al., 1985; Winterbourn & Kettle, 2013). TauCl is important for resolving inflammation and protecting neutrophils and surrounding tissues against production of the less specific HOCl (Peskin & Winterbourn, 2006). Because the life‐time of some oxidants is limited, we only focused on the earlier effects of oxidation in our experiments.

Solutions of p16 (20 μM) were exposed to increasing concentrations of each oxidant for 1 h to screen for an optimal oxidant concentration that would induce dimerization of p16 on a measurable time scale (Figure S2a–c). Hydrogen peroxide only reacts slowly and we used 200 μM since it has been previously shown to induce dimerization and amyloid formation of p16 (Heath, Gray, et al., 2024), and this concentration is approximately the upper limit in a cellular environment. This concentration was also used in the presence of 25 mM sodium bicarbonate (which is frequently reported as physiological level) (Winterbourn et al., 2023) to determine the differential effects of H_2_O_2_ and peroxymonocarbonate on the oxidation of p16. The final concentrations used for HOSCN and HOCl were 20 and 40 μM, respectively, and higher concentrations led to cleavage of the protein backbone, resulting in the formation of lower molecular weight fragments. The TauCl concentration was kept the same as HOCl for consistency. The oxidant concentrations and protein‐to‐oxidant ratios used throughout this study are presented in Table 1.

**TABLE 1:** Oxidant usage and conditions.

Oxidant	Final oxidant concentration used (*n*‐fold to protein concentration)	Sample buffer
Diamide	200 μM (10)	HEPES
Hydrogen peroxide (H_2_O_2_)	200 μM (10)	HEPES
Peroxymonocarbonate (H_2_O_2_ + HCO_3_^−^)	200 μM (10)	HEPES
Hypothiocyanous acid (HOSCN)	20 μM (1)	HEPES
Hypochlorous acid (HOCl)	40 μM (2)	Phosphate
Taurine Chloramine (TauCl)	40 μM (2)	Phosphate

**FIGURE 2:**
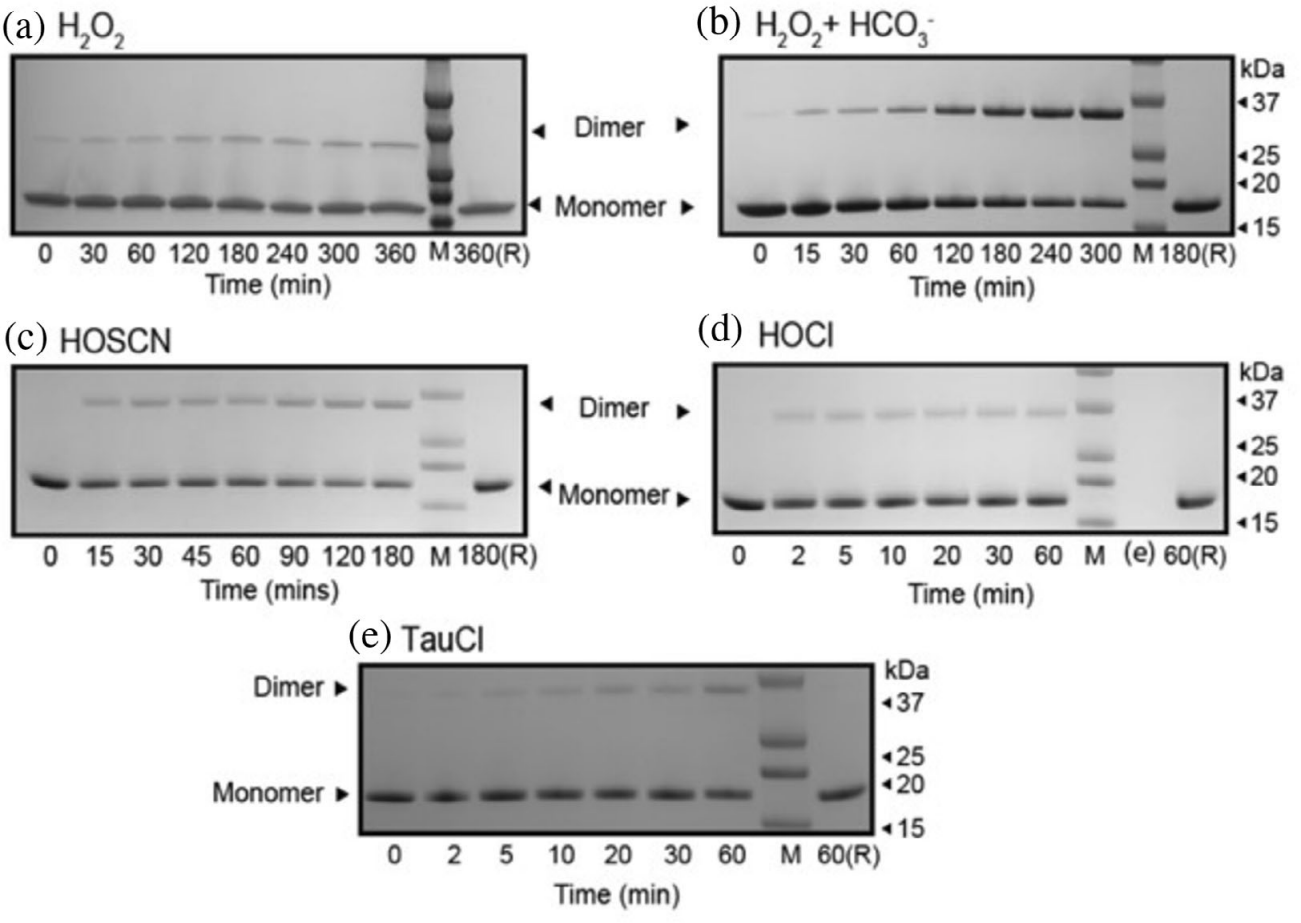
Dimerization of oxidized p16 and SDS‐PAGE analysis of 20 μM p16 oxidized during different time courses. Incubation over increasing time including (a) 200 μM hydrogen peroxide, (b) 200 μM hydrogen peroxide in the presence of 25 mM sodium bicarbonate (resulting in formation of peroxymonocarbonate), (c) 20 μM HOSCN, (d) 40 μM HOCl, and (e) 40 μM taurine chloramine. The last lane consists of oxidized samples including the addition of BME as a reducing agent (R).

p16 was treated with these concentrations of different oxidants for appropriate time points to explore their ability to induce protein dimerization (Figure 2). The samples were then subjected to NEM prior to SDS‐PAGE analysis. Treatment of p16 with 200 μM hydrogen peroxide for at least 30 min produced a weak dimer band that did not strongly increase even after 6 h of incubation (Figure 2a). In contrast, exposing p16 to 200 μM hydrogen peroxide in the presence of 25 mM sodium bicarbonate strongly increased the formation kinetics of the protein dimer band to an approximate half‐time of 4 h, as estimated from the intensity of the stained protein bands (Figure 2b). Peroxymonocarbonate is therefore a more efficient oxidizing agent of p16 than hydrogen peroxide alone. The cysteine‐specific oxidant HOSCN was also found to readily induce p16 dimerization upon treatment with 20 μM oxidant (Figure 2c). We further treated recombinant p16 samples with 40 μM HOCl and 40 μM taurine chloramine. HOCl is quick to induce dimers and lacks further time‐dependent increase in dimer band formation (Figure 2d). TauCl is slower to induce dimerization of p16 but shows a time‐dependent increase in dimers (Figure 2e). All samples contained fully reduced monomeric protein bands upon treatment with BME, indicating the presence of disulfide cross‐linked dimeric species. To investigate the details of the oxidative modifications, we next performed a mass spectrometry‐based analysis.

### 2.3 Oxidation causes p16 disulfide bond formation and other amino acid modifications

It has been previously shown that the Cys72 residue of p16 is essential for the formation of homodimers and amyloid fibrils, since variants lacking the cysteine residue do not form homodimers or amyloid fibrils upon oxidation (Göbl et al., 2020). We next confirmed the presence of dimeric p16 after oxidation by the various oxidants by mass spectrometry (Figure S3). All samples contained the dimeric species except for hydrogen peroxide, consistent with the weak dimer band observed by SDS‐PAGE and suggesting that dimer abundance was below the detection limit of the mass spectrometry method and the incubation conditions used. In addition to dimers, different species of monomers were present. Mostly, we identified unmodified p16, but also IAM‐bound protein and modifications such as sulfinic acid. This might explain the presence of monomeric species observed in the gel dimerization assays, where free and unmodified p16 could either be available for IAM alkylation prior analysis or the protein could be bound to early amyloid intermediates and therefore protected from cysteine oxidation.

To further investigate the covalent modifications, we performed a peptide analysis of p16 after exposure to the different oxidants. For this, oxidized p16 was spiked with unoxidized uniformly ^15^N‐labeled p16, then digested by the protease trypsin, and the resulting peptides were quantified using liquid chromatography–mass spectrometry (LC–MS). The ^14^N:^15^N ratio of each peptide was used to identify any p16 peptides that were substantially modified upon treatment with oxidants. The peptide sequences and their numbering can be found in Table 2 in the mass spectrometry methods section.

**TABLE 2:** Theoretical peptides of p16 upon full trypsin digestion.

Peptide	Position	Amino acid sequence	Monoisotopic mass (Da)
1	(−2)–22	(GA)MEPAAGSSMEPSADWLATAAAR	2347.06
2	23–24	GR^a^	231.13
3	25‐29	VEEVR	630.33
4	30–46	ALLEAGALPNAPNSYGR	1712.88
5	47–58	RPIQVMMMGSAR	1375.69
6	59–87	VAELLLLHGAEPNCADPATLTRPVHAAR	3049.58
7	88–89	EGFLDTLVVLHR	1397.77
8	100–103	AGAR^a^	373.21
9	104–107	LDVR	501.29
10	108–112	DAWGR	603.28
11	113–124	LPVDLAEELGHR	1347.71
12	125–128	DAVR^a^	459.24
13	129–131	YLR	450.26
14	132–138	AAAGGTR^a^	602.31
15	139–144	GSNHAR^a^	640.3
16	145–156	IDAAEGPSDIPD	1198.54

^a^ Peptides not detectable in the mass spectrometry analysis.

**FIGURE 3:**
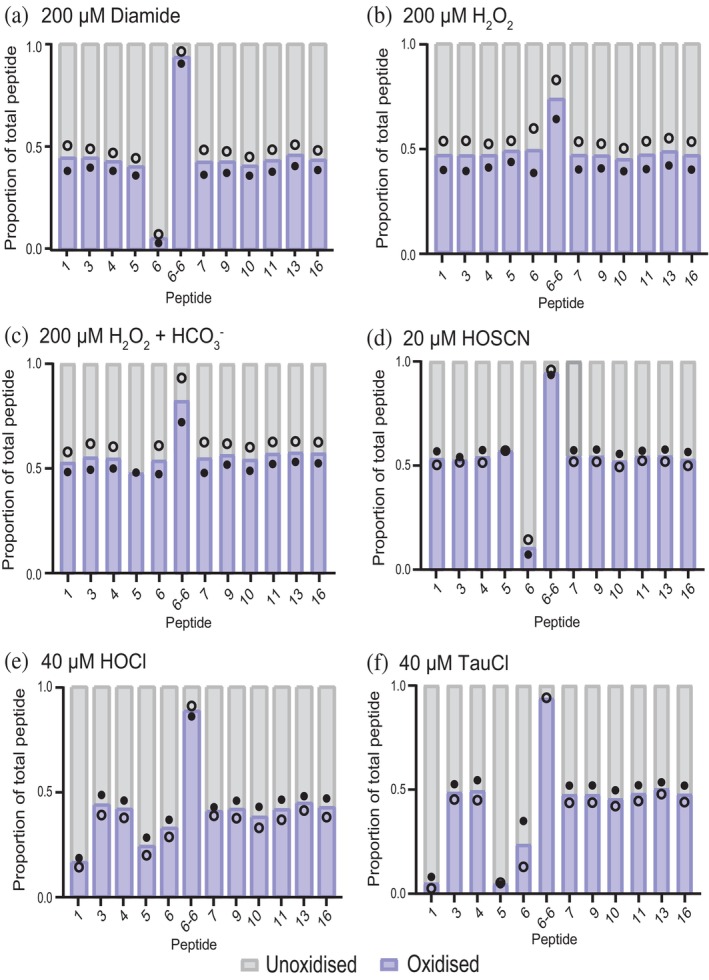
Modification of p16 peptides after treatment with various oxidants. (a) p16 was treated with diamide (3 h), (b) hydrogen peroxide alone (6 h), and (c) in the presence of 25 mM bicarbonate (peroxymonocarbonate, 3 h), (d) HOSCN (3 h), (e) HOCl (1 h), and (f) TauCl (1 h), followed by quenching of the reaction by NEM. Oxidized p16 was then spiked with unoxidized, isotopically labeled ^15^N p16 and digested overnight with trypsin at 25°C. LC–MS peptide analysis was performed to identify any covalent changes of each peptide. Averages of peptide peak areas for both the treated ^14^N‐labeled samples and ^15^N‐labeled p16 controls were plotted into stacked columns normalized to 100%, with closed and open circles representing the peak areas of each replicate (*n* = 2). In this analysis, any chemical modification results in a change in mass of the peptide, and therefore results in a loss in the treated ^14^N signal compared to the unoxidized ^15^N control.

When using the model oxidant diamide, the ratio between p16 peptides from the treated (^14^N‐labeled) and untreated (^15^N‐labeled) protein was essentially equal for all except the cysteine‐carrying peptide 6, indicating that these peptides were not modified, as expected for the cysteine‐specific oxidant. ^14^N‐labeled (treated) monomeric peptide 6, which harbors the cysteine residue, was barely detected (Figure 3a) with mainly dimeric peptide 6 being present and confirming the formation of an intermolecular disulfide bond upon oxidation with diamide. This increase in ^14^N‐labeled dimeric peptide 6 is seen after treatment by all oxidants tested (Figure 3a–f). Oxidation with hydrogen peroxide, peroxymonocarbonate, and the thiol‐specific oxidant HOSCN yielded similar results to diamide, with mainly disulfide bond formation observed (Figure 3b–d). For HOCl and TauCl, the ^14^N:^15^N ratios for some other peptides indicate additional modifications, in particular for peptides 1 and 5. Further analysis of the samples revealed several other oxidative modifications, as displayed in Table S1. We identified methionine sulfoxide formation on peptides 1 and 5 after treatment with either H_2_O_2_, peroxymonocarbonate, HOCl, or TauCl on multiple methionine residues. Chlorotyrosine was detected for one out of the two available tyrosine residues (Y44, peptide 4) after oxidation with HOCl. Peptide 10, which contains a tryptophan residue, showed a small decrease in the ^14^N:^15^N ratio in the HOCl and taurine chloramine treated samples (Figure 3e,f) and we observed the formation of hydroxytryptophan and *N*‐formylkynurenine, whereas no oxidation on the other tryptophan residue (W15, peptide 1) was identified. We note that these results cannot directly be compared to the dimer intensities observed by SDS‐PAGE in Figure 2 because they report different species (intact dimers *versus* individual tryptic peptides). In addition, peptide mass spectrometry analysis is subject to distinct analytical biases, including peptide digestion and ionization efficiencies, which only results in semi‐quantitative data.

**FIGURE 4:**
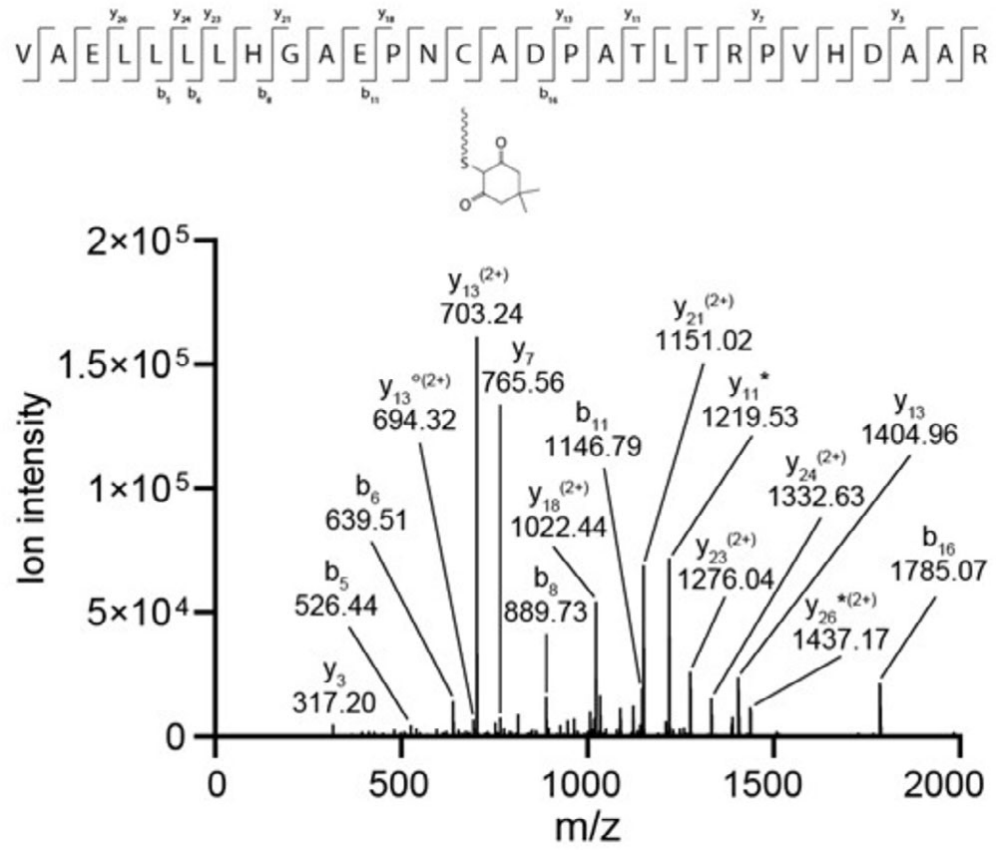
Incubation of p16 with hydrogen peroxide results in the formation of sulfenic acid. p16 (20 μM) was treated with hydrogen peroxide (200 μM) in the presence of dimedone (20 mM), then alkylated with iodoacetamide and digested with trypsin, before analysis by LC–MS/MS. Representative CID‐MS/MS spectrum for the [M + 3H]^3+^ ion of the VAELLLLHGAEPNC(dimedone)ADPATLTRPVHDAAR peptide (1064.21 *m*/*z*). The loss of water from a fragment ion is denoted by “°” where *y*_*x*_° is *y*_*x*_‐H_2_O. The loss of ammonia from a fragment ion is denoted by “*” where *y*_x_* is *y*_*x*_‐NH_3_.

We next characterized the details of the cysteine disulfide bond formation. The two most common mechanisms for their formation are disulfide exchange (Nagy, 2013) acting via nucleophilic substitution and oxidation via a reactive sulfenic acid intermediate (Winterbourn, 2008). We tested p16 for its ability to form a sulfenic acid intermediate when reacting with the physiological oxidants. The thiol‐specific compound 5,5‐dimethyl‐1,3‐cyclohexanedione (dimedone) is known to selectively react with sulfenic acid by forming a stable thioether (Gupta & Carroll, 2014). The detection of dimedone adducts therefore suggests the presence of sulfenic acid as a reaction intermediate. When performing the oxidation reaction with hydrogen peroxide under standard conditions in the presence of 20 mM dimedone, we indeed detected a dimedone adduct for the cysteine‐harboring peptide 6 (Figure 4) using mass spectrometry. The same dimedone adduct was found for each of the other physiological oxidants (Figure S4), confirming that disulfide bond formation can occur through sulfenic acid intermediates.

### 2.4 Kinetics of amyloid formation in the presence of different oxidants

**FIGURE 5:**
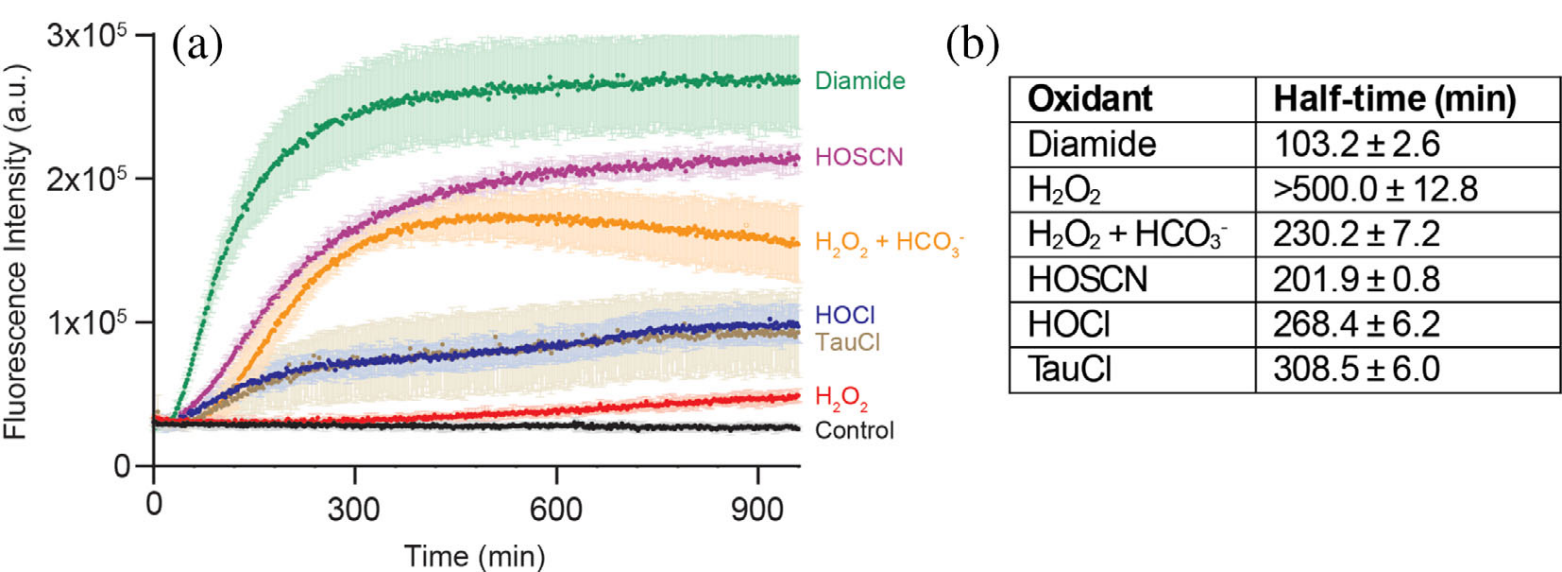
Thioflavin‐T fluorescence kinetics assay of p16 (20 μM) oxidized with oxidants at different concentrations. (a) p16 was incubated with 200 μM hydrogen peroxide (H_2_O_2_), 200 μM hydrogen peroxide in the presence of 25 mM sodium bicarbonate (H_2_O_2_ + HCO_3_^−^), 20 μM HOSCN, 40 μM HOCl, 40 μM HOCl which has been quenched by 16 mM methionine prior to the addition of the ThT dye, and 40 μM taurine chloramine. Samples were measured in triplicate and the standard deviation is presented. (b) Expansions on the right display the regions used for half‐time determination using AmyloFit (Meisl et al., 2016); individual replicates are plotted.

Next, we characterized the amyloid fibril formation of p16 when treated with the different oxidants using ThT fibril formation assays and kinetic analysis (Figure 5a). We first measured the stability of the ThT fluorescence dye in the presence of the oxidants using ^1^H NMR spectroscopy and we found that it was mainly stable but HOCl readily oxidized the reporter molecule (Figure S5). To avoid modification of the ThT dye by HOCl, methionine was added after treatment of p16 with HOCl for 10 min to scavenge any excess oxidant prior to the addition of the ThT dye and monitoring of amyloid formation. For the ThT fluorescence assay, following the treatment of the protein with 200 μM hydrogen peroxide there was very little increase in fluorescence, with an amyloid formation half‐time of >500 min, suggesting that there is little presence of ThT‐positive amyloid fibrils at the end of the experiment. However, when p16 was treated with hydrogen peroxide in the presence of bicarbonate (peroxymonocarbonate), there was a strong increase in fluorescence, with a clear plateau reached and a half time of amyloid formation of 230.2 ± 7.2 min. A similar sigmoidal increase in fluorescence could be seen when p16 was treated with HOSCN, showing an amyloid formation half‐time of 201.9 ± 0.8 min. The ThT assay of HOCl after quenching with methionine led to a slight increase in fluorescence, with a half time of amyloid formation of 268.4 ± 6.2 min. Similarly, when p16 is treated with taurine chloramine, there is a small increase in fluorescence, with a half time of 308 ± 6 min, although the latter two oxidants did not yield a clear and stable plateau (Figure 5b).

### 2.5 Visualization of the amyloid oxidation products by electron microscopy

**FIGURE 6:**
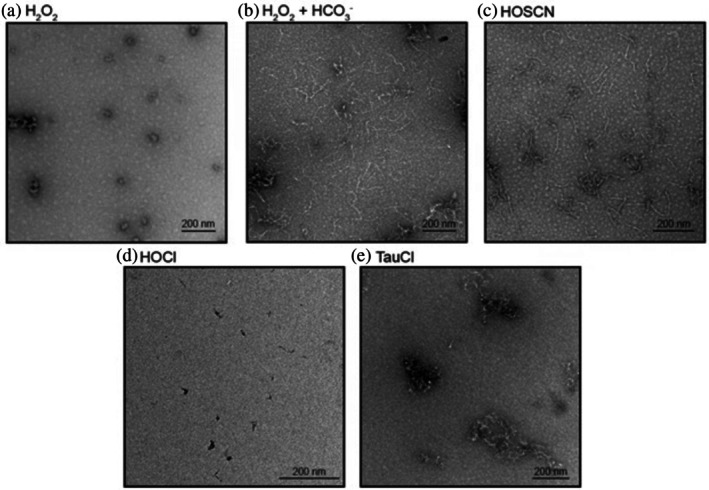
p16 forms amyloid fibrillar structures under specific oxidizing conditions. (a) Representative images of negative‐stain transmission electron microscopy of p16 treated with hydrogen peroxide (200 μM) alone and (b) with 25 mM bicarbonate, (c) 20 μM HOSCN, (d) 40 μM HOCl, (e) 40 μM TauCl for 48 h. Scale bars are present for the individual measurements.

Negative‐stain transmission electron microscopy was employed to examine morphological characteristics and formation of large structures of oxidized p16 after 48 h under standard oxidation conditions. Treatment of p16 with peroxymonocarbonate or HOSCN revealed the formation of short fibrillar structures that are characteristic of amyloid fibrils (Figure 6b,c), these structures closely resemble the amyloids formed by oxidation with diamide (Figure S1c). In contrast, oxidation with hydrogen peroxide alone, HOCl, or taurine chloramine revealed very little to no fibril‐like structures, but instead mainly resulted in the formation of amorphous protein aggregates (Figure 6a,d,e). These different morphologies likely reflect differences in the underlying dimerization mechanisms, reaction kinetics, or a combination of both factors.

## 3 DISCUSSION

The formation of amyloid fibrils is a major structural transition where repetitively aligned β‐strands assemble into long fibrillar aggregates (Chiti & Dobson, 2017). Protein amyloids are associated with a range of common diseases including Alzheimer's disease and Parkinson's disease, but specific fibrillar structures have also been discovered to be functional (Chiti & Dobson, 2017; Otzen & Riek, 2019). For a given protein, in vitro amyloid formation can result in different and distinct conformations; this phenomenon is referred to as polymorphism (Colletier et al., 2011; Tycko, 2015). It has been speculated that if an amyloid structure does not serve a biological function, it has not been evolutionary selected for this structural conformation, and the minima in the folding energy landscape can become relatively flat, permitting the formation of various conformations. High‐resolution structures of amyloids have provided outstanding insights such as different β‐sheet features present in reversible and irreversible amyloids (Frey et al., 2024), but the onset of amyloid formation is still poorly understood. It is generally thought that amyloids form through a primary nucleation event that can be of stochastic nature and highly dependent on the environment and conditions (Buell, 2022; Linse, 2019).

In contrast, the oxidation‐induced dimerization and transition into amyloid structures of p16 has previously been described to be highly reproducible when using the cysteine‐specific model‐oxidant diamide (Göbl et al., 2020; Heath, Gray, et al., 2024; Heath, Naughton, et al., 2024). This process is dependent on the oxidation of a single cysteine residue forming a regulatory, intermolecular disulfide bond that drives conformational transition. This study explores whether physiological oxidants, such as those involved in inflammation, are also capable of inducing this major structural conversion. We found that both peroxymonocarbonate and HOSCN are very efficient at converting p16 to the dimeric and amyloid states, implicating them as potential physiological oxidants for the structural transition and inactivation of p16.

All five tested oxidants were able to induce disulfide‐dependent dimerization of p16, but with varying rates. However, not all oxidants generated ThT‐positive fibrils with well‐defined amyloid morphology. Peroxymonocarbonate and HOSCN were very effective at converting p16 to the dimeric and amyloid states, producing structures closely resembling those generated by the model oxidant diamide, as highlighted by electron microscopy. In contrast, hydrogen peroxide alone and TauCl induced dimerization only slowly, reached low plateaus in ThT fluorescence assays, and showed no evidence of fibrillar structures by electron microscopy. While HOCl appeared relatively efficient to promote dimerization, it did not generate a typical single‐sigmoidal shaped ThT curve or extended fibrillar structures as detected by electron microscopy. High concentrations of HOCl were unable to form p16 homodimers possibly because of rapid formation of sulfinic acid, preventing the formation of disulfide bonds. Notably, peroxymonocarbonate‐ and HOSCN‐induced oxidation resulted in faster ThT kinetics while also leading to more ordered fibrillar structures. This distinction is further supported by the occurrence of non‐cysteine oxidation events. Both HOCl and taurine chloramine caused extensive oxidation of methionine, and these residues might play a structural role during the transition or in the final amyloid state. Indeed, we previously demonstrated that the triple‐methionine motif in peptide 5 (Met52, Met53, Met54) is essential for transition into amyloid and their side‐chain oxidation might alter amyloid properties (Heath, Naughton, et al., 2024). We also recently reported that reduction of the disulfide bond leads to disassembly of the amyloid structures (Heath, Gray, et al., 2024), and this process would be independent of the type of oxidant tested since all of them induced a cysteine‐dependent disulfide bond.

All physiological oxidants are able, to varying extents, to generate sulfenic acid intermediates. Peroxymonocarbonate was speculated to lead to sulfenic acid intermediates (Winterbourn et al., 2023) previously, and the mechanism of cysteine oxidation by HOSCN has also been shown to involve sulfenic acid intermediates (Skaff et al., 2009). Therefore, these intermediates seem to provide a common mechanistic entry point for p16 dimerization and subsequent transition into amyloids. Although it is not assessed here and it is unclear whether different oxidants generate structurally distinct p16 amyloids, our data show that they have characteristic effects on oxidation rates and to some extent, nature, and lifetime of intermediates involved in amyloid formation. In this context, the efficient transition into ordered amyloid structures induced by specific oxidants might indicate reduced polymorphism and could suggest a redox‐regulated functional structural switch.

p16 represents the first example of a protein that can reversibly convert between the monomeric and amyloid states through formation or reduction of a disulfide bond. This study highlights that innate differences between physiologically relevant oxidants strongly influence their ability to induce dimerization and amyloid formation of p16. Importantly, previous biochemical studies showed that p16 in the amyloid state is unable to inhibit its cellular targets, cyclin‐dependent kinases 4 and 6 (Göbl et al., 2020; Heath, Gray, et al., 2024). A detailed understanding of the chemical modifications and conformational changes that occur upon exposure of p16 to different oxidants is essential to understand such inactivation in a cellular environment.

## 4 METHODS

### 4.1 Protein expression and purification

An *Escherichia coli* codon‐optimized gene of *Homo sapiens* p16^INK4a^ (Uniprot‐ID: P42771) was cloned into a modified pETZ2 vector containing a kanamycin‐resistance gene (GenScript Biotech). The construct included an N‐terminal 6xHis tag followed by a protein A tag and a tobacco etch virus (TEV) protease cleavage site. The resulting wild‐type human p16 protein included an additional glycine–alanine sequence at the N terminus upon proteolytic cleavage. Chemically competent BL21(DE3) *E. coli* cells were transformed with the p16 harboring vector in the presence of kanamycin and stored as glycerol stock solutions at −80°C. Aliquots of these cells were grown in lysogeny broth (for unlabeled protein), or minimal media (for uniformly ^15^N‐labeled protein, containing ^15^NH_4_Cl as the only nitrogen source). Protein synthesis and purification was performed as reported in detail previously (Sethi et al., 2025). Briefly, the protein was expressed by induction with IPTG, the cells were lysed by sonication and the protein was isolated by nickel affinity chromatography and size exclusion chromatography and proteolytic digest of the tags. The buffer used for purification consisted of 20 mM HEPES, 110 mM potassium acetate, 5% (v/v) glycerol and 2 mM β‐mercaptoethanol at pH 8.0. The purified protein stock solution was aliquoted and stored at −80°C until further use. Mass spectrometry was employed to confirm that protein stocks were pure with unmodified amino acid residues. Before experiments, protein stocks were thawed on ice and freshly buffer exchanged into either 4‐(2‐hydroxyethyl)‐1‐piperazineethanesulfonic acid buffer (HEPES, 4 mM, pH 7.4) or phosphate buffer (sodium phosphate, 10 mM, pH 7.4) using an ÄKTA pure chromatography system (Cytiva) equipped with a desalting column (HiPrep 26/10 Desalting, Cytiva). The protein concentrations were measured using a NanoDrop 1000 spectrophotometer (ThermoFisher Scientific) using a computed extinction coefficient of 13,980 M^−1^·cm^−1^ and samples were concentrated to approximately 25 μM using centrifugal filter units (Amicon Ultra) with a 3 kDa molecular weight cut‐off.

### 4.2 Oxidation of p16

Oxidation of p16 protein was achieved by addition of different oxidants (diamide, hydrogen peroxide, hydrogen peroxide in bicarbonate buffer to yield peroxymonocarbonate, HOSCN, HOCl or taurine chloramine) at various concentrations (Table 1) to 20 μM monomeric p16. We found that HOCl oxidized the HEPES buffer (as confirmed by ^1^H nuclear magnetic resonance [NMR] spectroscopy, data not shown) and we used a 10 mM phosphate buffer at pH 7.4 instead. Taurine chloramine experiments were also performed in this buffer for better comparability. Sodium dodecyl sulfate polyacrylamide gel electrophoresis (SDS‐PAGE) was used to determine concentrations for each oxidant that would produce p16 dimers within the experimental timeframe (Figure S2). Samples were incubated with the chosen adequate concentrations of oxidant at room temperature (Table 1). Following incubation, 10 mM *N*‐ethylmaleimide (NEM) was added to all samples to prevent further modification of cysteine residues. Oxidation by HOCl and taurine chloramine was stopped by the addition of excess methionine (10 mM) prior to the addition of NEM (Kettle & Winterbourn, 1994). For gel electrophoresis time series experiments, all samples were timed to have the same set end point to ensure that all samples were handled the same following oxidation. For this, oxidant was added in a staggered fashion, with NEM treatment carried out at the same time for all samples.

A 5 mM stock solution of diamide was produced by dissolving powdered diamide (*N,N,N′,N′*‐tetramethylazodicarboxamide, Sigma Aldrich) in MilliQ water and stored at −20°C. All other oxidants were produced freshly and used within 6 h and were stored at 4°C during this time. Hydrogen peroxide stock solutions were produced by diluting an approximate 10 M stock solution (Fluka Analytical) in MilliQ water and confirming the concentration by measuring absorbance at 240 nm (Kettle & Winterbourn, 1994). Hydrogen peroxide was used as an oxidant either alone or in the presence of 25 mM sodium bicarbonate (Sigma Aldrich) (prepared in MilliQ water at a stock concentration of 125 mM and pH‐adjusted to 7.4 with HCl) to generate the oxidant peroxymonocarbonate. HOSCN was produced by incubating potassium phosphate buffer (10 mM, pH 6.6), sodium thiocyanate (Sigma Aldrich, 150 mM) and lactoperoxidase (Sigma Aldrich L2005, 2 mg/mL) with hydrogen peroxide (75 mM added in four 1‐min increments while vortexing). Lactoperoxidase was then removed by spinning the solution through a freshly washed 10 kDa molecular weight cut‐off centrifugal filter unit at 4°C (16,200 g for 7 min). The resulting concentration of HOSCN was determined by a TNB (5‐thio‐2‐nitrobenzoic acid) assay, measuring absorbance at 412 nm (Kettle & Winterbourn, 1994). The concentration of HOCl was determined by diluting bleach (Janola) as the HOCl stock, in MilliQ water 1:15, followed by a 1:10 dilution in 200 mM potassium hydroxide, measuring absorbance at 292 nm and using ε292 = 350 M^−1^ cm^−1^. Taurine chloramine (Sigma Alrich T8691) was produced by performing a 1:2 dilution of HOCl in 10 mM of taurine (made in phosphate buffer).

### 4.3 SDS‐PAGE electrophoresis

Oxidized samples were combined with SDS sample loading buffer with or without reducing agent (62.5 mM Tris base, 2% w/v SDS, 10% v/v glycerol, 0.01% w/v bromophenol blue, 100 mM β‐mercaptoethanol). Samples were then separated on a 4%–12% gradient polyacrylamide gel (NuPAGE Bis‐Tris Gels, Invitrogen) at room temperature and stained using Coomassie R250 staining solution.

### 4.4 Thioflavin T fluorescence assays

p16 samples (20 μM) were prepared in the presence of 10 μM Thioflavin T (ThT) dye, with oxidant being added immediately prior to the start of the measurement series. Following an initial 1 mm orbital shaking for 10 s, samples were excited at 435 nm, and the resulting fluorescence emission intensity was measured at 485 nm under quiescent conditions every 5 min. Samples were measured in triplicate in a 96‐well half‐area fluorescence plate (Corning CLS3881) on a SpectraMax ID3 microplate reader (Bio‐Strategy, PMT gain set to high). Amyloid formation half‐times were analyzed using the AmyloFit 2.0 web server (Meisl et al., 2016).

### 4.5 Solution NMR spectroscopy

For ThT stability testing, samples were prepared by treating 100 μM ThT dye with a five‐fold concentration of each oxidant to a final volume of 500 μL in different buffers (Table 1). Samples were incubated overnight at room temperature in a dark environment before measurement. The spectra were acquired on a Bruker 600 MHz spectrometer operated by an Avance III console equipped with a TXI triple‐resonance probe including gradients along the *z*‐axis. D_2_O (35 μL) was added prior to measurement for the lock signal. Samples were measured at 298 K using a one‐dimensional proton sequence employing water suppression through *z*‐gradient excitation sculpting. A total of 15,382 complex data points were acquired by accumulating 128 scans with an inter‐scan delay of 1 s. A single exponential convolution function of 2 Hz was applied to the data during Fourier transformation. All data were acquired, processed, and visualized using Topspin 3.6.5.

### 4.6 Negative‐stain transmission electron microscopy

Oxidized p16 (20 μM) was incubated for 48 h prior to making electron microscopy grids. Carbon/formvar‐coated copper grids (ProSciTech) were floated on a 7 μL droplet of protein sample for 60 s, then washed once with MilliQ water. Grids were then floated on a 7 μL drop of 2% (w/v) uranyl acetate stain for 30 s and were left to dry overnight. Micrographs were taken on a FEI Morgagni 268D transmission electron microscope at 80 kV.

### 4.7 Mass spectrometry analysis of oxidized p16

For the detection of full‐length protein and oxidative modifications, samples were treated with different oxidants (hydrogen peroxide and peroxymonocarbonate 5 h, HOSCN 3 h, HOCl and TauCl 1 h, diamide 3 h) followed by alkylation with iodoacetamide (50 mM).

For experiments investigating sulfenic acid formation, p16 was treated with oxidants as above (except for diamide) in the presence of dimedone (Sigma Aldrich D153303, 20 mM), followed by alkylation with iodoacetamide (Sigma Aldrich I1149, 50 mM).

For experiments investigating the modification of p16 peptides, p16 was treated with oxidants as described above. Oxidized p16 (unlabeled) was then mixed with an equal amount of unoxidized, uniformly isotopically ^15^N‐labeled p16.

For peptide analysis, samples were digested overnight with trypsin using a 50:1 protein:trypsin weight ratio at 25°C and subsequently analyzed by HPLC‐coupled nanospray tandem mass spectrometry (MS/MS) using a Thermo Scientific Velos Pro ion trap mass spectrometry coupled to a Dionex Ultimate 3000 HPLC system. Samples were stored on the autosampler tray at 5°C prior to injection using a 50 μL injection loop. The temperature of the heated capillary was 275°C. Separation of the digested protein samples was performed on a reverse phase column (Jupiter 4 μM Proteo 90 Å column 150 × 2.0 mm). Water containing 0.1% formic acid was used as Solvent A, and acetonitrile containing 0.1% formic acid was used as Solvent B. The column was equilibrated with 95% Solvent A and 5% Solvent B for 5 min before a linear gradient was run for 20 min to 55% Solvent A and 45% Solvent B in order to achieve separation. The column was then flushed with 5% Solvent A and 95% Solvent B for 5 min and subsequently re‐equilibrated in the initial conditions for 5 min. A flow rate of 0.2 mL/min was used throughout. The eluted tryptic peptides were then analyzed using a data‐dependent nth order double play scan procedure. For this, the 10 most abundant peaks in a full scan (300–2000 *m*/*z*) were sequentially selected and a MS/MS scan was performed using collision‐induced dissociation with a normalized collision energy of 40%. MS/MS spectra were recorded using dynamic exclusion with a repeat count of 3, a repeat duration of 30 s, and an exclusion duration of 60 s. The data was analyzed using Thermo Xcalibur Qual Browser 4.2.47, with chromatogram and peak area data processed using the Genesis detection algorithm. Peptide fragments were manually assigned based on Roepstorff–Fohlman nomenclature (Roepstorff & Fohlman, 1984) (Table 2).

For intact protein analysis, whole protein samples were analyzed using anAccucore‐15‐C4 HPLC column (50 mm × 2.1 mm, 2.6 μm; Thermo Fisher Scientific). The column temperature was set to 60°C. The column was equilibrated with 90% Solvent A and 10% Solvent B for 2.5 min and then a linear gradient was run for 2.1 min to 20% Solvent A and 80% Solvent B to elute the proteins. The column was then flushed with 20% Solvent A and 80% Solvent B for 2.5 min and re‐equilibrated at the initial condition for 2.5 min. A flow rate of 0.4 mL/min was used, and 1 μg of protein was injected for each sample. Mass spectral data were acquired from 3 to 9 min of each chromatographic separation, scanning between *m*/*z* 410 and 2000 in positive‐ion mode at a normal scan rate. Spectra were averaged over the full length of each protein peak using Thermo Xcalibur Qual Browser 4.2.47 (Thermo Fisher Scientific) and deconvoluted to yield the molecular masses using ProMass for Xcalibur (Version 2.8 rev. 5; Novatia LLC).

## AUTHOR CONTRIBUTIONS

**Nicholas J. Magon:** Conceptualization; investigation; writing – review and editing; visualization; validation. **Christoph Göbl:** Conceptualization; investigation; data curation; formal analysis; project administration; visualization; writing – review and editing; writing – original draft. **Sarah G. Heath:** Investigation; formal analysis. **Aakriti Sethi:** Investigation; formal analysis. **Shelby G. Gray:** Investigation. **Briana R. Smith:** Investigation; writing – review and editing; writing – original draft; data curation; formal analysis; visualization. **Vanessa K. Morris:** Investigation; conceptualization; data curation; writing – review and editing.

## FUNDING INFORMATION

This study was funded by Marsden Fund project 21‐UOO‐128 (CG) and 24‐UOO‐179 managed by Royal Society Te Apārangi, and Canterbury Medical Research Foundation project MPG2021‐Morris (VKM) and MPG2022‐Goebl (CG).

## Supporting information


**Figure S1:** Diamide‐induced oxidation of p16 forms homodimers and subsequent amyloid fibrils. p16 (20 μM) was oxidized with diamide (200 μM). (a) SDS‐PAGE of p16 oxidized for subsequent time points. The homo‐dimeric band appears after oxidation and is reduced after the addition of the reducing agent BME (R). (b) Unnormalized thioflavin‐T fluorescence kinetics assay of p16 with and without oxidizing agent measured in triplicate; the data were normalized for determination of the half‐times. (c) Negative‐stain transmission electron micrograph of oxidized p16 after 48 h oxidation; the scale bar corresponds to 100 nm.
**Figure S2:** Concentration‐dependent dimerization of oxidized p16 after 1 h of incubation. p16 was incubated with (a) HOSCN, (b) HOCl, and (c) TauCl.
**Figure S3:** Mass spectrometry analysis of oxidized p16 in the absence (a) and presence (b–g) of the different oxidants.
**Figure S4:** Incubation of p16 with a range of oxidants results in the formation of sulfenic acid. p16 in the presence of 20 mM dimedone was treated with (a) 200 μM hydrogen peroxide (H_2_O_2_), (b) 200 μM hydrogen peroxide in the presence of 25 mM sodium bicarbonate (HCO_4_^−^), (c) 40 μM hypochlorous acid (HOCl), (d) 40 μM taurine chloramine (TauCl), or (e) 20 μM hypothiocyanous acid (HOSCN). Samples were subsequently incubated with 50 mM iodoacetamide to alkylate any free cysteine residues, before being analyzed by intact protein LC/MS. Spectra recorded over the full width of the protein peak were averaged and deconvoluted. The deconvoluted spectra are representative of two to three separate experiments. Inset graphs show the 16,500–17,000 Da region.
**Figure S5:** Oxidation of the Thioflavin T dye by different oxidants. (a) ^1^H NMR reference spectrum of ThT. (b) ThT after exposure of various oxidants displayed in different colors; no modifications were observed and peak reduction is a sole result of sample dilution. (c) ThT exposure to HOCl leads to various oxidative modifications and bleaching of the fluorescent dye.
**Table S1:** Modified peptides identified by LC/MS after treatment of p16 with a range of oxidants. p16 (20 μM) was treated with 200 μM hydrogen peroxide (H_2_O_2_), 200 μM hydrogen peroxide in the presence of 25 mM sodium bicarbonate (HCO_4_^−^), 40 μM hypochlorous acid (HOCl), 40 μM taurine chloramine (TauCl) or 20 μM hypothiocyanous acid. Samples were subsequently incubated with 50 mM iodoacetamide to alkylate any free cysteine residues, and digested with trypsin, before analysis by LC–MS/MS. * = modified residue.

## Data Availability

The data that support the findings of this study are available from the corresponding author upon reasonable request.
