# Supplementary material for: The impact of physiological oxidants on triggering amyloid formation of the cell cycle regulator protein p16INK4A
Source: Protein Sci. 2026 Jul 15;35(8):e70723. doi: 10.1002/pro.70723 (PMC13373305; doi:10.1002/pro.70723)
Supplement: Supplementary file 1 — Figure S1: Diamide‐induced oxidation of p16 forms homodimers and subsequent amyloid fibrils. p16 (20 μM) was oxidized with diamide (200 μM). (a) SDS‐PAGE of p16 oxidized for subsequent time points. The homo‐dimeric band appears after oxidation and is reduced after the addition of the reducing agent BME (R). (b) Unnormalized thioflavin‐T fluorescence kinetics assay of p16 with and without oxidizing agent measured in triplicate; the data were normalized for determination of the half‐times. (c) Negative‐stain transmission electron micrograph of oxidized p16 after 48 h oxidation; the scale bar corresponds to 100 nm. Figure S2: Concentration‐dependent dimerization of oxidized p16 after 1 h of incubation. p16 was incubated with (a) HOSCN, (b) HOCl, and (c) TauCl. Figure S3: Mass spectrometry analysis of oxidized p16 in the absence (a) and presence (b–g) of the different oxidants. Figure S4: Incubation of p16 with a range of oxidants results in the formation of sulfenic acid. p16 in the presence of 20 mM dimedone was treated with (a) 200 μM hydrogen peroxide (H2O2), (b) 200 μM hydrogen peroxide in the presence of 25 mM sodium bicarbonate (HCO4 −), (c) 40 μM hypochlorous acid (HOCl), (d) 40 μM taurine chloramine (TauCl), or (e) 20 μM hypothiocyanous acid (HOSCN). Samples were subsequently incubated with 50 mM iodoacetamide to alkylate any free cysteine residues, before being analyzed by intact protein LC/MS. Spectra recorded over the full width of the protein peak were averaged and deconvoluted. The deconvoluted spectra are representative of two to three separate experiments. Inset graphs show the 16,500–17,000 Da region. Figure S5: Oxidation of the Thioflavin T dye by different oxidants. (a) 1H NMR reference spectrum of ThT. (b) ThT after exposure of various oxidants displayed in different colors; no modifications were observed and peak reduction is a sole result of sample dilution. (c) ThT exposure to HOCl leads to various oxidative modifications and bleaching of the fluo [file PRO-35-e70723-s001.docx]

Supplementary Information

**The impact of physiological oxidants on triggering amyloid formation of the cell cycle regulator protein p16^ink4a^**

**Briana R. Smith**^1^**, Nicholas J. Magon**^1^**, Aakriti Sethi**^1^**, Shelby G. Gray**^2^**, Sarah G. Heath**^1^**, Vanessa K. Morris**^2,3^**, Christoph Göbl**^1,3^*****

^1^ Mātai Hāora - Centre for Redox Biology and Medicine, Department of Pathology and Biomedical Science, University of Otago, Christchurch, New Zealand

^2^ School of Biological Sciences, University of Canterbury, Christchurch, New Zealand

^3^ Biomolecular Interaction Centre, University of Canterbury, Christchurch, New Zealand

* to whom correspondence should be submitted, [christoph.goebl@otago.ac.nz](mailto:christoph.goebl@otago.ac.nz)


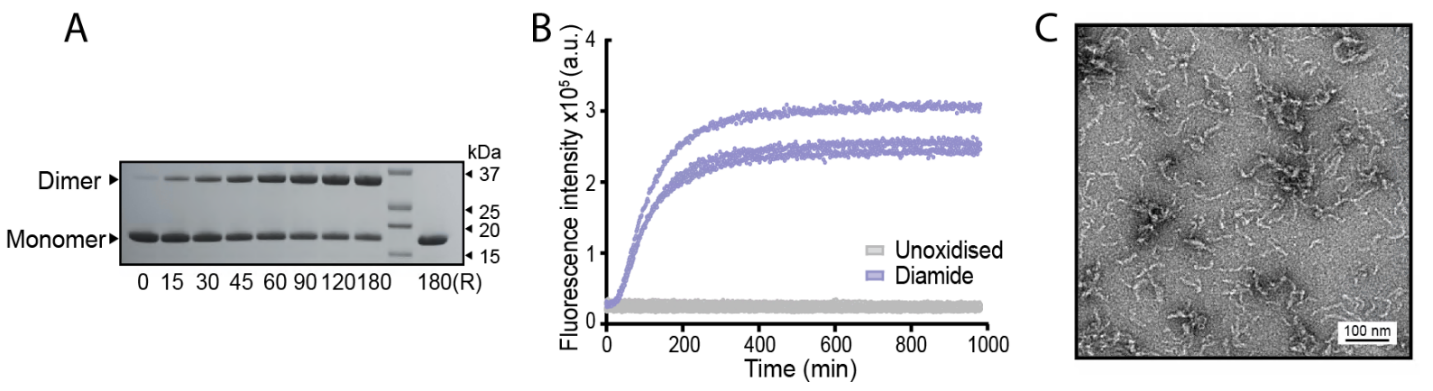
 **Supplementary Figure S1: Diamide-induced oxidation of p16 forms homodimers and subsequent amyloid fibrils.**p16 (20 μM) was oxidised with diamide (200 μM). **A.** SDS-PAGE of p16 oxidised for subsequent time points. The homo-dimeric band appears after oxidation and is reduced after the addition of the reducing agent BME (R). **B.** Unnormalised thioflavin-T fluorescence kinetics assay of p16 with and without oxidising agent measured in triplicate; the data were normalised for determination of the half-times. **C.** Negative-stain transmission electron micrograph of oxidised p16 after 48 h oxidation; the scale bar corresponds to 100 nm.

**Supplementary Figure S2: Concentration-dependent dimerisation of oxidised p16 after 1 hour of incubation.** p16 was incubated with **A.** HOSCN, **B.** HOCl and **C.** TauCl.
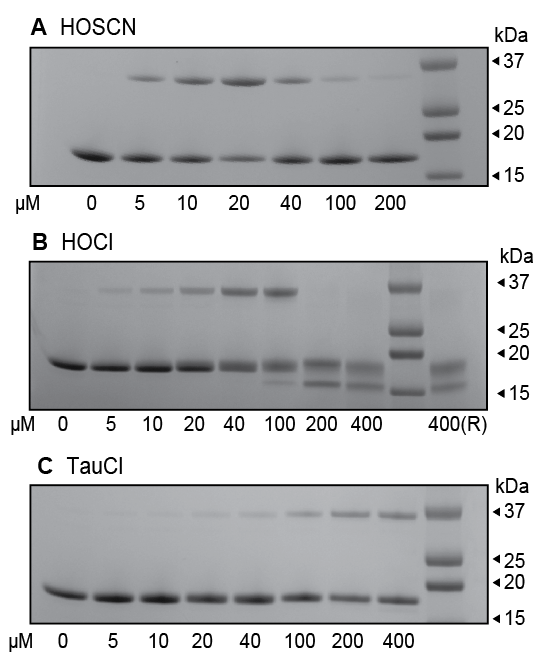


**Supplementary Figure S3: Mass spectrometry analysis of oxidised p16 in the absence (A.) and presence (B.-G.) of the different oxidants.**
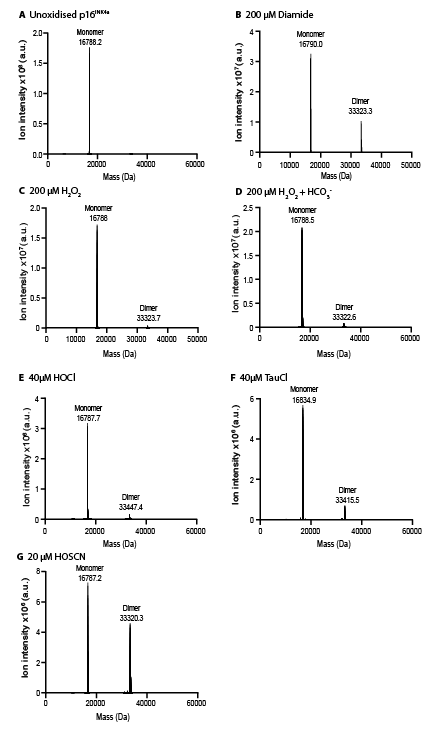


| **Tryptic peptide** | **Modification** | **Residues** | **m/z (charge state)** | **Oxidant** |
| --- | --- | --- | --- | --- |
| GAM*EPAAGSSMEPSADWLATAAAR | Methionine sulfoxide | M1 | 1182.95 (+2) | H_2_O_2_, HCO_4_^-^ |
| GAMEPAAGSSM*EPSADWLATAAAR | Methionine sulfoxide | M9 | 1182.95 (+2) | H_2_O_2_, HCO_4_^-^ |
| GAM*EPAAGSSM*EPSADWLATAAAR | Methionine sulfoxide | M1, M9 | 1190.86 (+2) | H_2_O_2_, HCO_4_^-^, HOCl, TauCl |
| ALLEAGALPNAPNSY*GR | ^35^Chlorotyrosine | Y44 | 874.74 (+2) | HOCl |
| RPIQVMM*MGSAR | Methionine sulfoxide | M53 | 697.00 (+2) | H_2_O_2_, HCO_4_^-^, HOCl, TauCl |
| RPIQVMM*M*GSAR | Methionine sulfoxide | M53, M54 | 705.11 (+2) | H_2_O_2_, HCO_4_^-^, HOCl, TauCl |
| RPIQVM*M*M*GSAR | Methionine sulfoxide | M52, M53, M54 | 705.11 (+2) | HCO_4_^-^, HOCl, TauCl |
| DAW*GR | Hydroxytryptophan | W110 | 310.81 (+2) | HOCl, TauCl |
| DAW*GR | *N*-Formyl-  kynurenine | W110 | 318.67 (+2) | HOCl, TauCl |

**Supplementary Table 1. Modified peptides identified by LC/MS after treatment of p16 with a range of oxidants.** p16 (20 µM) was treated with 200 µM hydrogen peroxide (H_2_O_2_), 200 µM hydrogen peroxide in the presence of 25 mM sodium bicarbonate (HCO_4_^-^), 40 µM hypochlorous acid (HOCl), 40 µM taurine chloramine (TauCl) or 20 µM hypothiocyanous acid. Samples were subsequently incubated with 50 mM iodoacetamide to alkylate any free cysteine residues, and digested with trypsin, before analysis by LC-MS/MS. * = modified residue.

**
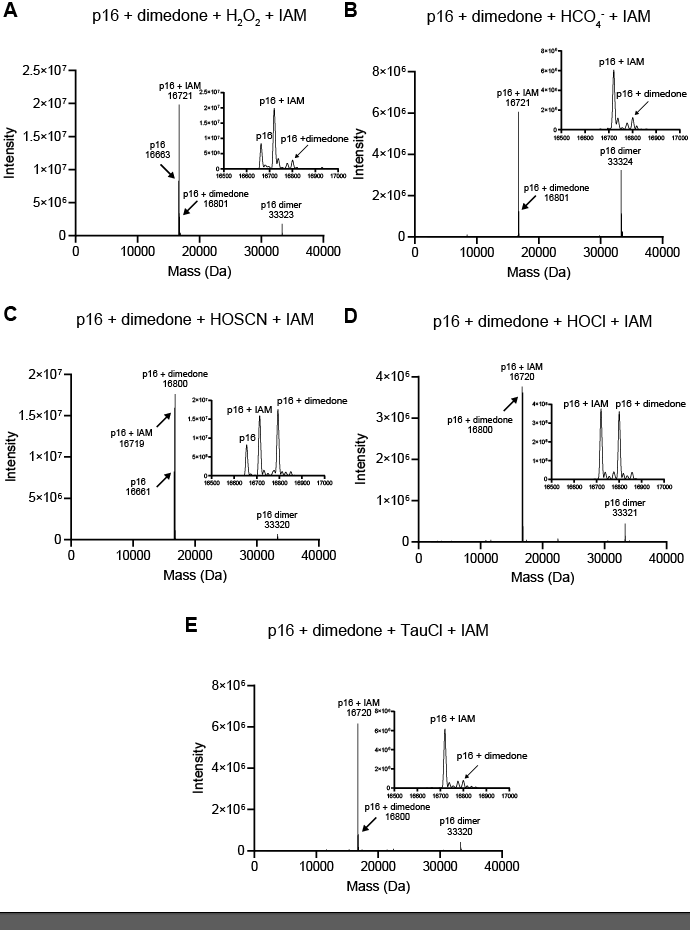
**

**Supplementary Figure S4: Incubation of p16 with a range of oxidants results in the formation of sulfenic acid.** p16 in the presence of 20 mM dimedone was treated with **A.** 200 µM hydrogen peroxide (H_2_O_2_), **B**. 200 µM hydrogen peroxide in the presence of 25 mM sodium bicarbonate (HCO_4_^-^), **C**. 40 µM hypochlorous acid (HOCl), **D**. 40 µM taurine chloramine (TauCl), or **E**. 20 µM hypothiocyanous acid (HOSCN). Samples were subsequently incubated with 50 mM iodoacetamide to alkylate any free cysteine residues, before being analysed by intact protein LC/MS. Spectra recorded over the full width of the protein peak were averaged and deconvoluted. The deconvoluted spectra are representative of two-three separate experiments. Inset graphs show the 16500-17000 Da region.


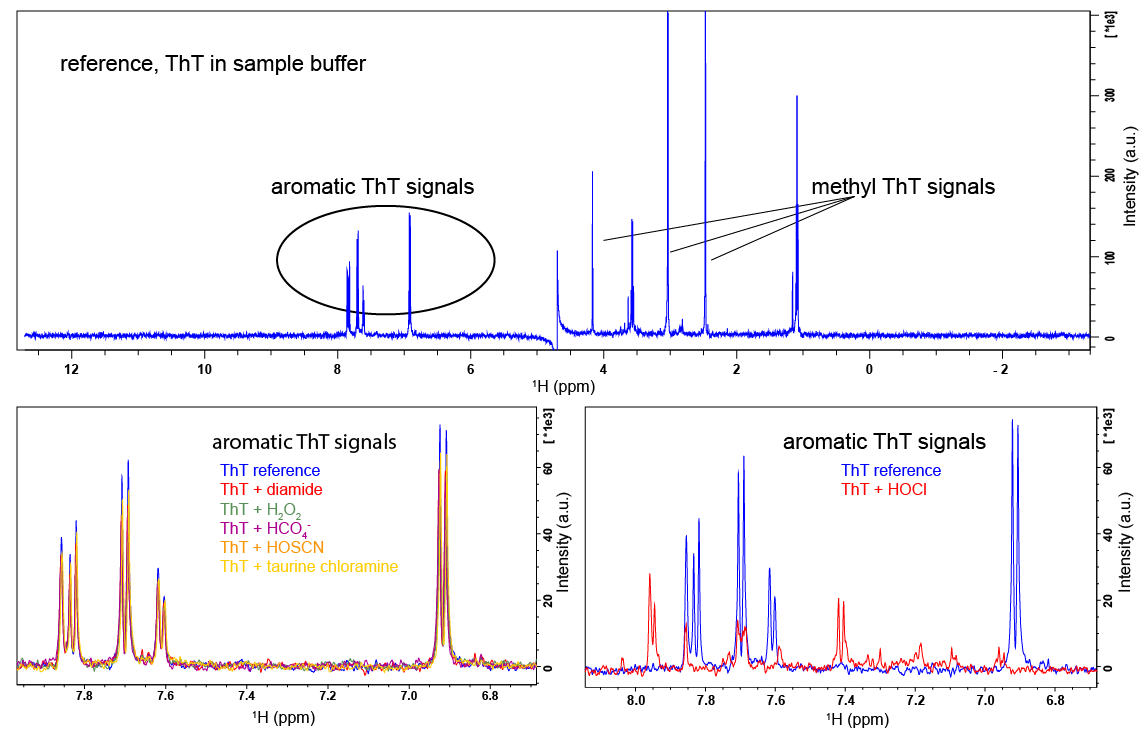


**Supporting Figure S5: Oxidation of the Thioflavin T dye by different oxidants. A.** ^1^H NMR reference spectrum of ThT. **B.** ThT after exposure of various oxidants displayed in different colours; no modifications were observed and peak reduction is a sole result of sample dilution. **C.** ThT exposure to HOCl leads to various oxidative modifications and bleaching of the fluorescent dye.
